# 介孔共价有机骨架核壳固定相的制备及其用于黄芪中黄芪甲苷含量的分析

**DOI:** 10.3724/SP.J.1123.2025.09020

**Published:** 2026-06-08

**Authors:** Xingyun ZHAO, Jiangyan JIN, Xiaojian LIU, Lijuan YAN, Siyu YANG, Zhenwei ZHANG, Liyun ZHANG, Rongfang WU

**Affiliations:** 1.山西大学中医药现代研究中心，化学生物学与分子工程教育部重点实验室，山西 太原 030006; 1. Modern Research Center for Traditional Chinese Medicine，Shanxi University，the Key Laboratory of Chemical Biology and Molecular Engineering of Ministry of Education，Taiyuan 030006，China; 2.山西白求恩医院（山西医学科学院），山西医科大学第三医院，同济山西医院，山西 太原 030032; 2. Shanxi Bethune Hospital，Shanxi Academy of Medical Sciences，the Third Hospital of Shanxi Medical University，Tongji Shanxi Hospital，Taiyuan 030032，China

**Keywords:** 介孔, 共价有机骨架, 固定相, 黄芪甲苷, mesoporous, covalent organic framework （COF）, stationary phase, astragaloside Ⅳ

## Abstract

中药质量控制一直是中药现代化研究领域的重点和难点，中药成分复杂，对先进分离材料的研究提出了更高的需求。共价有机框架材料（COF）是一类由多齿有机单元通过共价键连接而成的新型多孔晶体材料，在催化与色谱分析等领域具有良好的应用价值。本研究创新性地构建了一种基于共价有机框架的新型核壳型色谱固定相。以2，4，6-三（4-氨基苯基）-1，3，5-三嗪（TAPT）和1，4-苯二甲醛（TA）为构筑单元，在聚合诱导胶体聚集（PICA）法制备的二氧化硅微球表面，通过多步聚合策略成功制备了TAPT-TA-COF@SiO₂核壳复合材料。系统表征结果表明，所制备的固定相具有优异的单分散性，COF层在SiO₂核表面呈现均匀包覆。氮气吸附-脱附等温线分析证实材料具有典型的介孔结构，比表面积和孔径分布与原始多孔SiO_2_微球相近，为高效传质提供了结构基础。红外表征说明出现新材料C=N的特征吸收峰1 515 cm^-1^，粉末X射线衍射也表明TAPT-TA-COF@SiO₂复合材料具有COF的晶体衍射峰。色谱性能评价显示，该固定相在反相色谱模式下，通过疏水相互作用、*π-π*相互作用以及独特的介孔结构，实现了中等极性和非极性混合物的基线分离。制备重复性实验结果表明，TAPT-TA-COF@SiO₂填充色谱柱的批内相对标准偏差（RSD）低于1.6%，展现出优异的制备稳定性。此外，在中药质量控制应用方面，该方法在黄芪甲苷含量测定中显示出一定优势，测得含量为0.085%，符合药典标准要求。本研究不仅为COF基色谱固定相的设计与制备提供了新的技术路径，而且为分离科学与药学领域的交叉深入研究奠定了坚实基础。

## Abstract (en)

Quality control of traditional Chinese medicine （TCM） has always been a key and challenging issue in the field of its modernization research. This has posed a high demand for advanced separation materials due to the complex compositions. Covalent organic framework materials （COFs） are a new class of porous crystalline materials composed of multidentate organic units connected by covalent bonds. They have demonstrated significant application value in areas such as catalysis and chromatographic analysis. This study focused on developing a novel core-shell-type chromatographic stationary phase using 2，4，6-tris（4-aminophenyl）-1，3，5-triazine （TAPT） and 1，4-benzenedicarboxaldehyde （TA） as building units. The TAPT-TA-COF@SiO_2_ core-shell composite materials were successfully prepared on the surface of silica microspheres using a multi-step polymerization strategy， in which the SiO_2_ cores were fabricated using the polymerization-induced colloidal aggregation （PICA） method. The imine-linked TAPT-TA-COF@SiO_2_ core-shell stationary phase was subjected to comprehensive physicochemical characterization and chromatographic evaluation experiments. The analytical techniques employed included scanning electron microscopy （SEM）， transmission electron microscopy （TEM）， energy dispersive spectroscopy analysis （EDS）， nitrogen adsorption-desorption isotherms， Fourier transform infrared spectroscopy （FT-IR）， and powder X-ray diffraction （PXRD）. The systematic characterization results clearly indicate that the prepared stationary phase exhibits excellent monodispersity， and the COF layer is uniformly coated on the surface of the SiO₂ core. TEM characterization demonstrated that the thickness of the COF materials on the surface of SiO_2_ is approximately 110 nm. Furthermore， FT-IR spectra were collected and the results demonstrated that the characteristic stretching vibrations at 3 209， 2 927， and 1 515 cm^-1^， attributed to N-H， C-H， and C=N stretching， confirm the condensation reaction between TAPT and TA. In the XRD pattern， the peaks observed at 16.6°， 18.9°， 25.2° and 27.5° were attributed to the COF material and were consistent with previous reports， thereby confirming the successful synthesis of this derivative. The N_2_ adsorption-desorption isotherm analysis confirmed that the material possesses a typical mesoporous structure. Its specific surface area and pore size distribution are similar to those of the original porous SiO₂ microspheres， subsequently providing a structural basis for efficient chromatographic mass transfer kinetics. In addition， the chromatographic performance was investigated. And it was confirmed that the stationary phase was effectively used for the separation of representative neutral polar or non-polar compounds such as benzenes， alkylbenzenes， phthalate esters， formamides， and aniline mixtures. These compounds are separated due to hydrophobic interactions， *π-π* interactions， and the unique mesoporous structure in the reversed-phase chromatography mode. ACN-water （30∶80 or 40∶60， volume ratio） was selected as the mobile phase at a flow rate of 1 mL/min. The results of the methodological validation indicate that the intra-batch relative standard deviations （RSDs） of one TAPT-TA-COF@SiO₂ packed chromatographic column were less than 1.6%， demonstrating excellent preparation reproducibility. Furthermore， this stationary phase was applied to the quality control of traditional Chinese medicine. Specifically， it was used to determine the content of astragaloside Ⅳ in *Astragalus* according to pharmacopoeial records. The measured result met the pharmacopoeia standard of ≥0.08%. This research work not only provides new opportunities for advancing fundamental and applied research on novel COF stationary phases， but also helps to further promote in-depth research on the application of COF materials at the intersection of separation science and pharmaceutical sciences.

黄芪为豆科植物蒙古黄芪（*Astragalus membranaceus* （Fisch.） Bge. var. *mongholicus* （Bge.） Hsiao）或膜荚黄芪（*A. membranaceus* （Fisch.） Bge.）的干燥根，具有补气固表、利水消肿、敛疮生肌的功效^［1］^。黄芪甲苷（astragaloside Ⅳ）作为黄芪中特征性的羊毛酯醇型四环三萜皂苷类化合物，具有调节免疫、抗炎、抗肿瘤等作用^［2，3］^，也是含有黄芪的中医经典名方及现代复方制剂质量控制与优劣评价的核心检测指标，其含量测定结果直接关系到药品的安全性、有效性和质量一致性^［4］^。高效液相色谱（HPLC）技术已成为中药质量控制中最常用的分析检测技术。然而，中药基质的复杂性以及待测组分结构的多样性，对色谱分离固定相提出了更高的要求，固定相需要同时兼顾对不同极性、不同结构类型化合物的分离能力，这对固定相的孔径分布、表面化学性质以及机械稳定性都提出了更高的技术要求。因此，具有稳定和合适介孔的功能化固定相的设计和制备一直是高度复杂和结构多样性样品分离分析研究的核心^［5］^。

共价有机框架材料（COFs）作为一类具有长程有序结构的多孔晶体有机聚合物，因其结构可控与功能可调，在材料科学领域广受关注^［6］^。通过精准调控构筑单元的化学组成与共价连接方式，可实现COF材料的定向制备，为其功能化设计提供了灵活路径。目前，COFs有多种制备方法和类型，包括聚芳醚基COFs^［7］^、硼酸衍生的COF材料^［8，9］^、*β*酮胺连接COF（TpBD^［10］^）、亚胺连接COF（LZU-1^［11，12］^和CTpPa-1^［13］^）、腙连接COF^［14］^等。COFs材料普遍具备高比表面积、精确可调的孔道结构及丰富的功能位点等核心特征，被广泛应用于传感器^［14］^、抗菌^［15］^、催化^［16，17］^领域。在分离科学领域，COFs因多样性网络结构、孔隙结构和较高表面积等特点，为色谱分离材料提供了理想的选择^［18］^。COFs已被用作吸附剂^［19，20］^，以及气相色谱^［21，22］^、毛细管电色谱^［23］^、高效液相色谱^［24］^和手性LC色谱^［25］^的新型分离材料。此外，灵活的框架结构还为合成后修饰提供了充足空间，通过在框架中引入特定功能基团，可定向赋予COFs新的物理化学特性，进一步拓展其在分离科学领域的应用^［26］^。例如在样品前处理中，Lin等^［27］^采用多元表面自组装策略制备了离子（2-溴乙基）三甲基溴化铵共价有机骨架表面接枝单片吸附剂，通过Williamson反应制备固相萃取柱成功实现了复杂基质中马兜铃酸的高效富集。在气相色谱分离中，Yan等^［28］^合成了2，2′-双（三氟甲基）联苯胺官能团单体，制备了三氟甲基功能化二维共价有机骨架，用于异构体的高分辨率气相色谱分离。在液相色谱中，Yuan等^［29］^通过接枝（*S*）-2-甲基吡咯烷到共价有机骨架上制备了核壳复合CCOF-TpPa-Py@SiO_2_手性柱，该手性柱对多种外消旋化合物表现出良好的拆分性能。上述已报道的工作均表明COFs提供了更稳定、更多样的色谱应用。然而，目前使用的COFs多数是二维材料，或没有规则形态。根据van Deemter方程^［30］^，直接填充不规则形状的固定相会导致色谱柱效率低、峰形差和选择性差。尽管已制备了具有优异选择性和良好峰形的球形COF固定相^［31］^，仍然存在一些问题，比如由于COFs色谱柱压力高，制备长色谱柱具有挑战性，以及COFs色谱柱效率通常较低等^［32］^。因此，开发制备工艺可控、性能更优异的COFs固定相仍是当前色谱材料领域的研究重点，同时可满足中药及复杂样品的分离需求。

基于此，本研究以2，4，6-三（4-氨基苯基）-1，3，5-三嗪（TAPT）和1，4-苯二羧醛（TA）为构筑单元在SiO_2_球载体上通过逐层表面聚合制备了亚胺连接COFs。并对其开展系统性色谱性能评价：选取单取代苯类化合物、多环芳烃（PAHs）、磺胺嘧啶、苯胺类及邻苯二甲酸盐等标准物质混合液，考察材料对不同结构类型化合物的分离能力。同时将其应用于黄芪中黄芪甲苷含量的测定分析，实验结果显示，方法精密度和稳定性良好，为中药质量控制提供了一种可行的方法，方法相较于文献报道的检测体系具有一定优势^［4］^。本研究成功实现了COF在SiO₂载体表面的均匀包覆，制备过程中未对多孔SiO₂的孔道结构造成堵塞，且有效消除了纯COF材料微孔结构对传质效率的不利影响。充分证实了均匀包覆型COF@SiO₂材料用于黄芪中黄芪甲苷含量的分析时，该方法简便、灵敏、准确，在中药分析领域具有良好的应用前景。未来可进一步设计合成亲水型、离子型等功能化COF色谱基微球，针对性解决药学分析、组学研究等领域中复杂样品的前处理难题，满足实际应用需求。

## 1 实验部分

### 1.1 仪器

JSM-5600扫描电子显微镜（SEM，日本东京JEOL公司）；JEOL JEM-2000 EX透射电子显微镜（TEM，日本电子株式会社有限公司）；Autosorb iQ2吸附仪（美国康塔仪器公司）；DZF型真空干燥箱（北京科伟永兴仪器有限公司）。TENSOR27傅里叶变换红外光谱仪（德国Bruker公司），SmartLab-9kw X射线粉末衍射仪（PXRD，日本Rigaku公司）。高效液相色谱系统由两个P230高压恒流泵和一个紫外检测器组成（日本岛津公司）；Rheodyne 7725i手动进样阀与定量环（上海屹利科学仪器有限公司）；DG230-2在线脱气器（依利特（大连）分析仪器有限公司）；ELSD-UM 5800蒸发光检测器，配备UMA-10LP空气发生器（上海通微分析科技有限公司）。

### 1.2 试剂与材料

本研究用黄芪为恒山黄芪，经山西大学中医药现代研究中心秦雪梅教授鉴定，其中恒山黄芪基原是蒙古黄芪的干燥根。对照品黄芪甲苷（纯度≥98%，批号：HA012079198，宝鸡市辰光生物科技有限公司）。平均粒径为10~20 nm的硅溶胶购自德州晶火无机材料商店。不锈钢色谱柱管（150 mm×4.6 mm）购自依利特（大连）分析仪器有限公司。硫脲、甲苯、乙苯、丙苯、丁苯、甲酰胺、*N，N*-二甲基甲酰胺、*N，N*-二乙基甲酰胺、2-氯苯胺盐酸盐、1-萘胺、邻苯二甲酸二甲酯、邻苯二甲酸二乙酯、邻苯二甲酸二环己酯和邻苯二甲酸二辛酯（分析纯，上海麦克林生化科技有限公司）。尿嘧啶、苯丙酮、芴、苊、蒽、菲、芘、盐酸苯胺、1-苯丙醇、磺胺嘧啶、磺胺胍、磺胺、磺胺二甲基嘧啶和磺胺噻唑（分析纯，上海阿拉丁试剂有限公司）。氨丙基三乙氧基硅烷（APTES）（分析纯，美国Sigma-Aldrich公司），乙腈（HPLC级，赛默飞世尔科技（中国）有限公司），TAPT（纯度99%）和TA（纯度99%）购自国药化学试剂有限公司。

### 1.3 实验方法

#### 1.3.1 亚胺TAPT-TA-COF微球的制备

参照文献［^33^］，采用聚合诱导胶体聚集法（PICA）制备二氧化硅微球。然后称取2.2 g二氧化硅微球，分散在40 mL 5%盐酸溶液中，在110 ℃下回流12 h，将其3 000 r/min离心2 min，再用水洗涤2次、甲醇洗涤1次并在真空条件下干燥。取上述酸化二氧化硅微球和2 mL APTES分散于约30 mL甲苯中，组成混合物在110 ℃下回流24 h，3 500 r/min离心2 min，再用甲醇洗涤3次并在真空条件下干燥，获得NH_2_-SiO₂。最后将改性后二氧化硅微球分散在40 mL乙腈中，分3批次加入70.8 mg TAPT、40.2 mg TA、4.5 mL乙酸三者的混合物，在60 ℃下反应3 d。最后，通过3 000 r/min离心2 min，再用水洗涤3次、甲醇洗涤1次并在真空条件下干燥，获得TAPT-TA-COF@SiO₂微球。合成路线示意如图1所示。

**图1:**
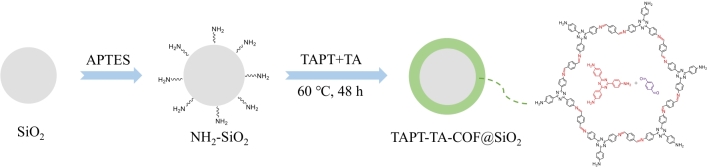
TAPT-TA-COF@SiO_2_材料的制备流程图

#### 1.3.2 色谱柱制备方法

采用文献［^34^］报道的匀浆法进行色谱柱填充，并针对本研究微球特性对工艺参数略作调整，具体步骤如下：将所制备的微球3 g悬浮分散于30 mL甲醇-异丙醇（1∶1，体积比）的混合溶剂中，超声5 min混匀倒入匀浆罐中。在40 MPa下放置10 min，然后压力降到20 MPa再放置20 min，最后压力降为0。在此条件下填充到150 mm×4.6 mm规格色谱柱中。

#### 1.3.3 供试品溶液的制备

对照品溶液的制备：取黄芪甲苷对照品适量，精密称定，加入80%甲醇水溶液制成质量浓度为3 mg/mL的黄芪甲苷对照品溶液。

供试品溶液的制备：称取黄芪粉末5 g加蒸馏水煎煮2次，第1次加10倍量的水，煎煮1.5 h，16层纱布过滤药渣，第2次再加8倍量的蒸馏水煮沸1 h、过滤。合并2次滤液，放冷后加入无水乙醇至乙醇体积分数为75%，密闭置4 ℃冰箱中静置24 h后取上清液减压浓缩至稠膏状，回收乙醇，膏液于70 ℃鼓风干燥箱中干燥至恒重得黄芪浸膏。称取黄芪浸膏溶于超纯水，混匀制成2.50 g/mL的黄芪溶液，4 ℃保存备用。

#### 1.3.4 分析条件

采用TAPT-TA-COF@SiO_2_核壳固定相液相色谱柱（150 mm×4.6 mm，5 µm），流动相：乙腈-水（35∶65，体积比），流速为l mL/min，柱温为25 ℃；蒸发光检测器检测参数：空气流速为2.5 L/min，管温保持在80 ℃。

## 2 结果与讨论

### 2.1 TAPT-TA-COF@SiO_2_微球的表征

#### 2.1.1 电镜与能谱表征分析

为观察材料是否固载到硅胶上，首先对合成的材料进行了扫描电镜和透射电子显微镜分析。如图2所示，所有材料均表现出高度单分散和均匀的纳米尺寸，COF成功均匀涂覆在硅球表面。通过SEM测量粒径范围约3~6 μm，可以看出TAPT-TA-COF@SiO_2_表面与裸SiO_2_表面不同，几乎没有观察到其他明显的COF纳米球或碎片，透射电镜可以看到SiO_2_微球表面存在约为114 nm厚度的COF壳层。因此，以上表征说明纳米COF材料成功固载到硅胶表面。

**图2:**
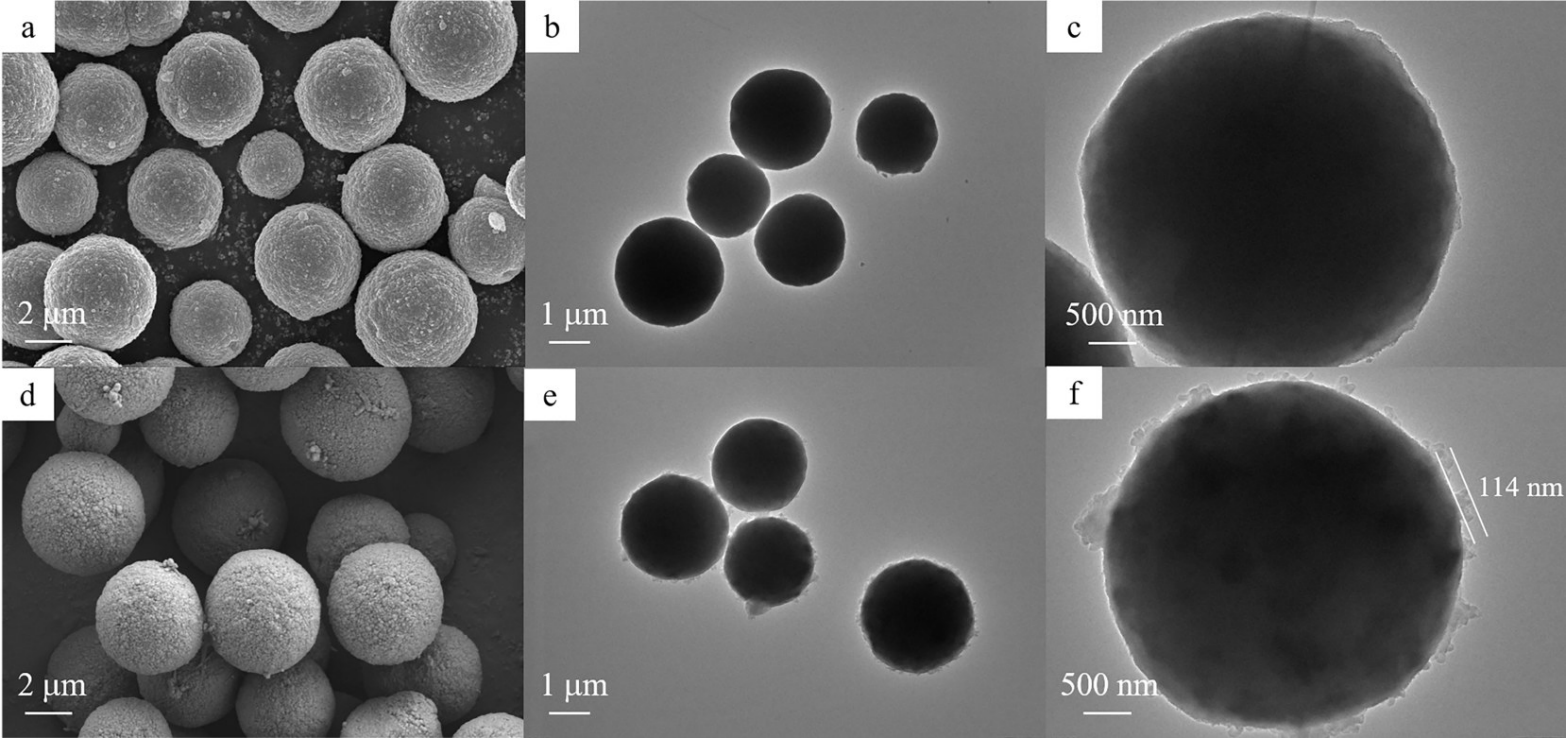
（a~c）SiO_2_和（d~f）TAPT-TA-COF@SiO_2_材料的SEM及TEM图

进一步，通过能量散射谱（EDS）表征研究了TAPT-TA-COF@SiO_2_的C、N、O和Si的空间元素分布。元素分布结果如图3所示，所有元素均匀地分布在二氧化硅表面。此外，COF上存在的特征元素N，测定的N元素所占的原子百分比和重量百分比分别为8.55%和6.96%（见表1）。因此，该测定结果更形象地表明了COF网络均匀固定在SiO_2_表面。

**图3:**
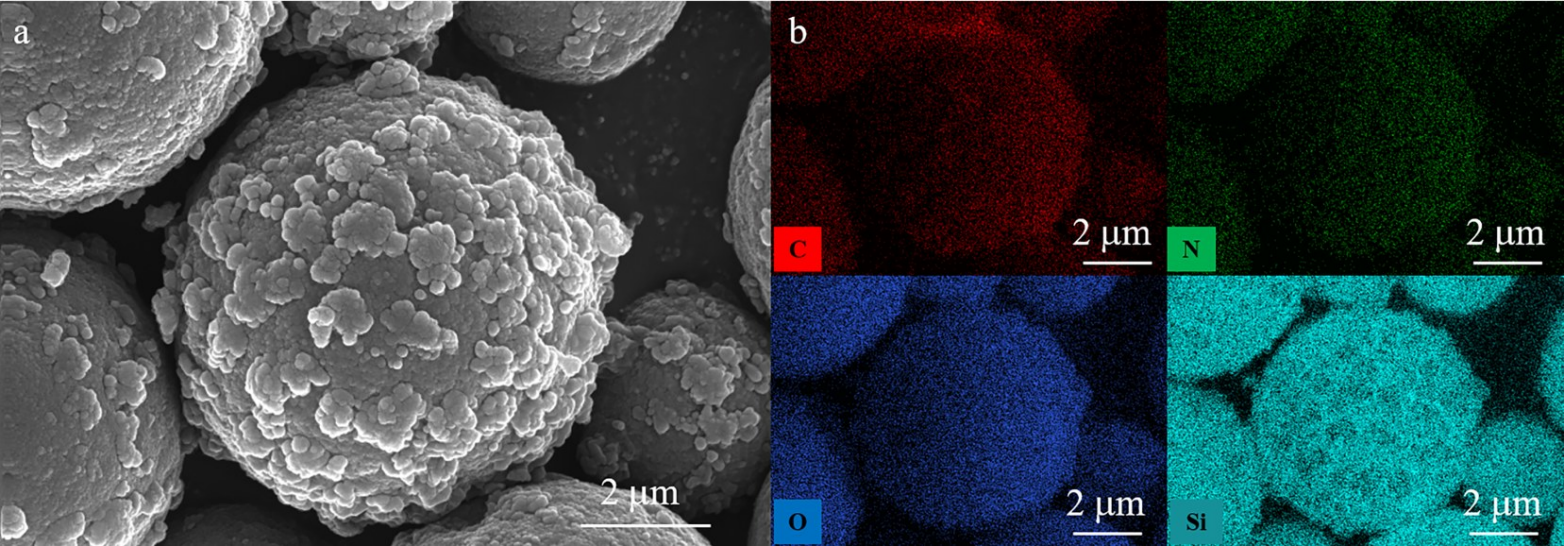
TAPT-TA-COF@SiO_2_的（a）SEM图像和（b）元素空间分布图

**表1:** TAPT-TA-COF@SiO_2_的元素含量

Element	Weight/%	Atom/%
C	22.77	32.64
N	6.96	8.55
O	33.95	36.54
Si	36.32	22.27

#### 2.1.2 氮气吸附测试

为测试材料的比表面积及孔径大小，进行氮气吸附实验，结果见图4。初始二氧化硅微球的比表面积为204 m^2^/g，孔径为9.2 nm。TAPT-TA-COF@SiO_2_比表面积约为164 m^2^/g，孔径7.6 nm，相比二氧化硅微球，其比表面积、孔体积和孔径均有所减小。说明COF材料在二氧化硅微球上成功键合。且本工作制备的材料具有独特的介孔孔径，适用于一般有机化合物分离。

**图4:**
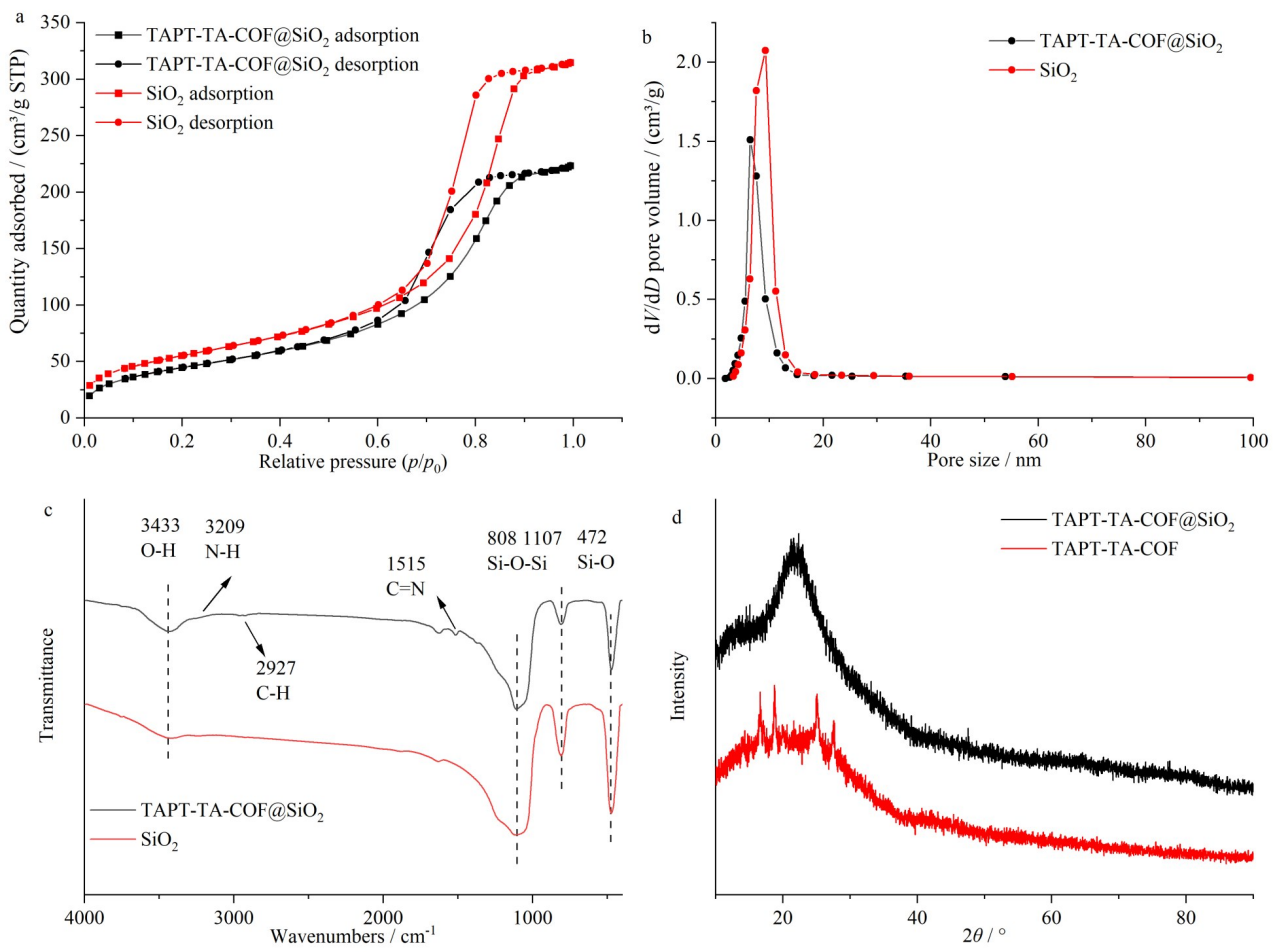
SiO_2_、TAPT-TA-COF@SiO_2_材料的（a）氮气吸附脱附曲线、（b）孔径分布图、（c）红外表征图与（d）TAPT-TA-COF、TAPT-TA-COF@SiO_2_材料的PXRD表征图

#### 2.1.3 红外光谱与Ｘ射线粉末衍射分析

采用红外光谱对合成的SiO_2_和TAPT-TA-COF@SiO_2_进行了表征（图4c）。TAPT-TA-COF@SiO_2_微球的红外光谱同时呈现出二氧化硅和TAPT-TA-COF的特征伸缩振动。3 433、1 107、808和472 cm^-1^处的峰归因于二氧化硅的O-H振动、Si-O-Si反对称振动、Si-O-Si对称拉伸吸收和Si-O弯曲振动吸收峰^［35］^，比裸二氧化硅响应略有降低。此外，在3 209、2 927、1 515 cm^-1^位置的特征吸收峰可能源于N-H^［36］^、C-H^［24］^及TAPT和TA缩合形成的C=N振动吸收峰^［24］^，说明TAPT-TA-COF@SiO_2_微球同时出现了N-H、C-H和C=N特征吸收峰，即醛基和氨基成功缩合形成COF材料包裹在二氧化硅外层。

为进一步证明TAPT-TA-COF是否固载到硅胶上，采用X射线粉末衍射对单独合成的TAPT-TA-COF材料与TAPT-TA-COF@SiO_2_复合微球进行了表征。如图4d所示，合成的TAPT-TA-COF存在一个10°~40°宽的弥散峰外，还有明显的很多尖锐结晶峰。即在16.6°、18.9°、25.2°和27.5°处有4个弱峰，属于COF的特征衍射峰。对于复合结构，SiO_2_峰更明显，且没有尖锐的结晶峰残留，说明基本都是SiO_2_非晶网格骨架构成，微球复合结构在大致2*θ*=13°附近有一个小的弥散峰对应于COF类材料，在10~40°附近的非晶弥散峰，这也说明复合微球里存在COF材料的结构特点，表明COF成功修饰在硅球上。

### 2.2 TAPT-TA-COF@SiO_2_材料色谱柱分离实验

#### 2.2.1 反相色谱保留机制考察

在流动相为乙腈-水（35∶65、30∶70、25∶75，体积比）、柱温为30 ℃、流速为1 mL/min的色谱条件下，用苯系物和烷基苯混合物样品来考察TAPT-TA-COF@SiO_2_液相色谱柱的反相保留机制。结果表明，随着乙腈体积分数从25%增至35%，乙苯的保留因子降低，丙苯和丁苯之间的分离度减小。因此，这些非极性化合物在该色谱柱上表现出典型的反相液相色谱（RPLC）保留机制，即烷基链长度从甲苯增加到正丁基苯，4种化合物的洗脱顺序与其疏水性一致，见图5a。此外，如图5b所示，疏水性较强的苯乙酮、甲苯、乙苯、芴等分析物在流动相乙腈体积分数为25%、30%和35%的条件下实现分离，且峰形效果良好，芴是二苯并五环，因其疏水性最强在相同流动相条件下保留时间最长，也表明了COF柱的反相分离性能。在本实验中，这些化合物在制备的色谱柱上的理论塔板数处于同等水平。

**图5:**
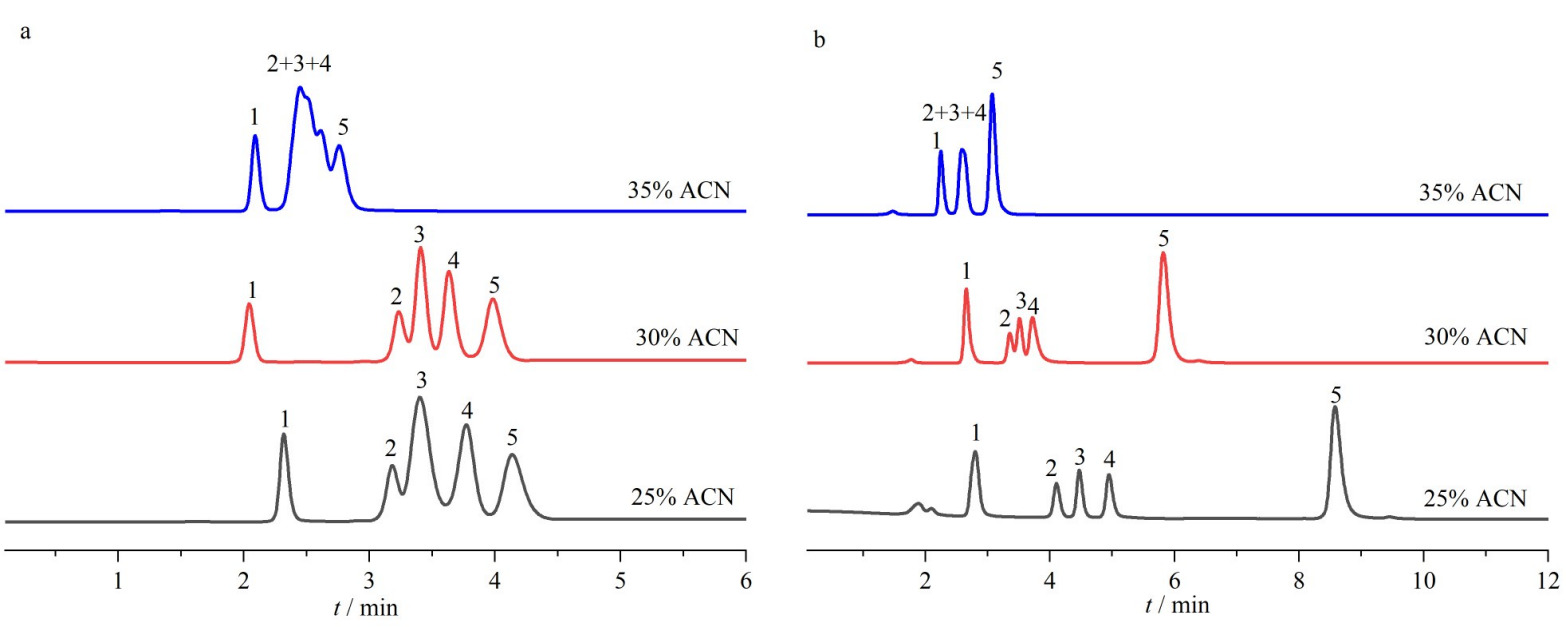
在流动相中含有不同体积分数的乙腈时TAPT-TA-COF@SiO_2_色谱柱对（a）苯系物和（b）烷基苯分离的色谱图

#### 2.2.2 色谱柱的重复性、稳定性和色谱柱效率

在流动相为乙腈-水（30∶70，体积比）、柱温为30 ℃、流速为1 mL/min的色谱条件下，用苯系物和烷基苯混合物样品来考察TAPT-TA-COF@SiO_2_液相色谱柱的重复性。同一支TAPT-TA-COF@SiO_2_色谱柱经过3次重复测试的色谱图见图6a和6b。采用同一支TAPT-TA-COF@SiO_2_色谱柱时，苯系物和烷基苯保留时间的RSD分别低于1.6%和1.4%。

**图6:**
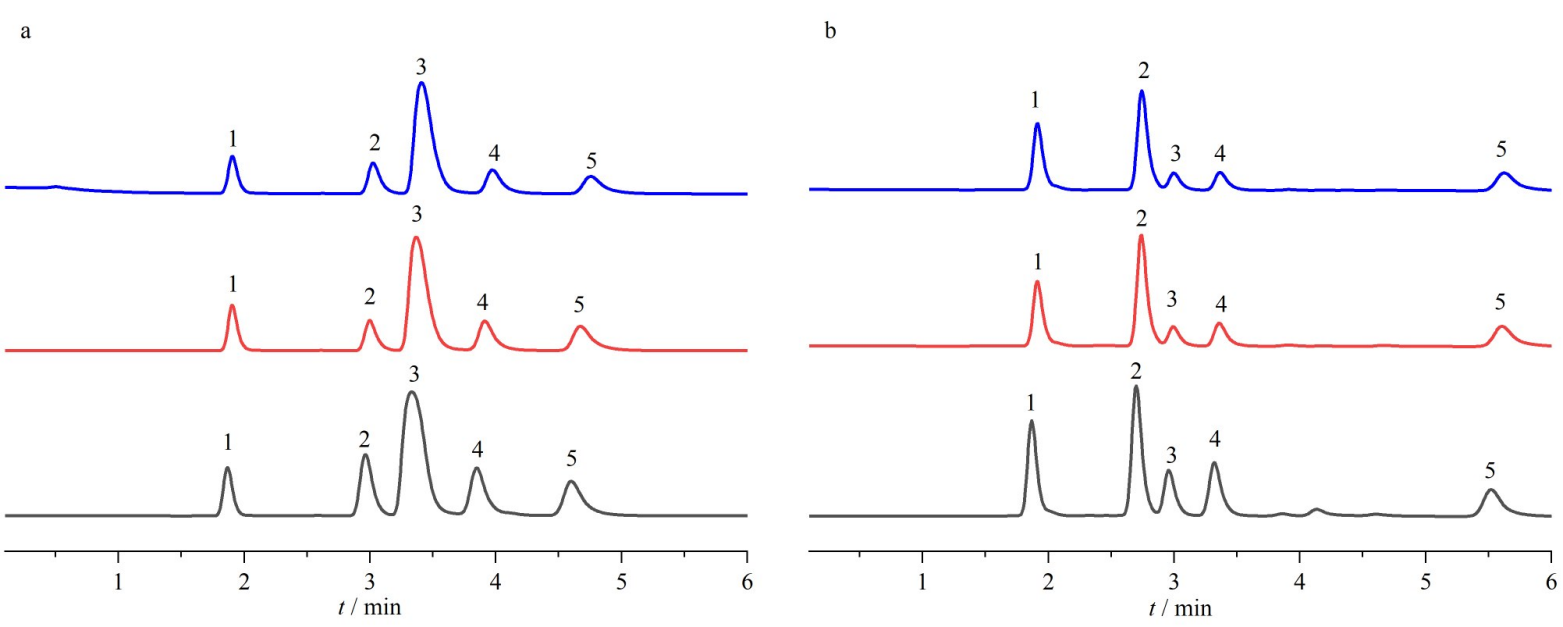
TAPT-TA-COF@SiO_2_色谱柱对（a）苯系物和（b）烷基苯的分离重复性考察

接着考察了其色谱分离柱效，在最佳流速1 mL/min、流动相为乙腈-水（30∶70，体积比）条件时，TAPT-TA-COF@SiO_2_微球填充柱上芴的柱效约为40 000 N/m。该数据与实验室装填相同粒径商品C18填料色谱柱的柱效数据相近，说明制备的材料可以用作固定相。

#### 2.2.3 色谱柱的分离性能考察

杂质沼气厂样品及发酵残渣中磺胺类与四环素类抗生素的精准定性定量分析，对环境污染物溯源、生态风险评估及沼气生产过程的安全生产监控具有重要现实意义。因此，本研究在流动相为乙腈-水（30∶70、25∶75或20∶80，体积比）、柱温为30 ℃、流速为1 mL/min的色谱条件下，测试了磺胺嘧啶、磺胺胍、磺胺、磺胺二甲基嘧啶和磺胺噻唑5个物质的混合物供试样品在TAPT-TA-COF@SiO_2_色谱柱上的分离效果。如图7a所示，在流动相乙腈-水为20∶80（体积比）条件下实现了基线分离，磺胺甲嘧啶与磺胺噻唑色谱分离度最高，且峰形良好。如图7b所示，在流动相组成乙腈-水为20∶80~30∶70（体积比）时，5种化合物在TAPT-TA-COF@SiO_2_色谱柱上保留因子随乙腈体积分数增加而减小。因此，实验表明合成的COF材料具有弱疏水保留特点，可进行磺胺嘧啶类化合物的分离分析。

**图7:**
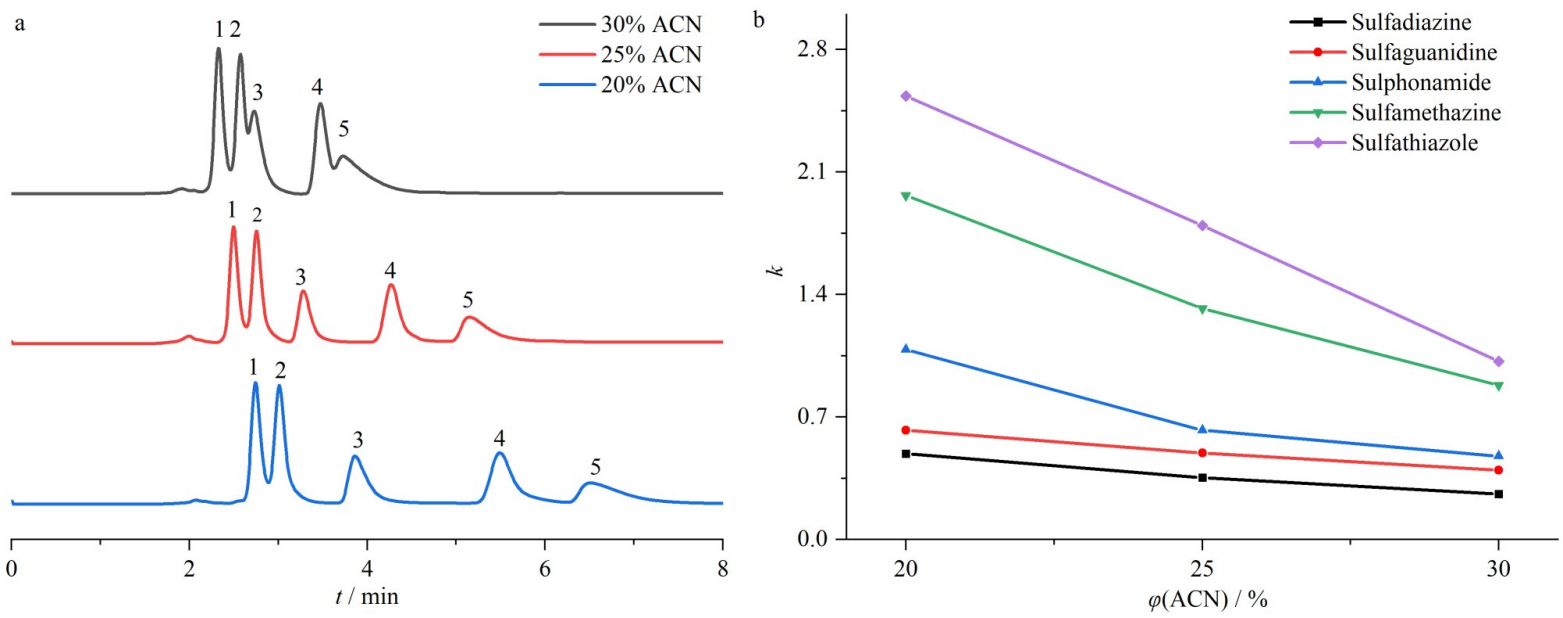
采用TAPT-TA-COF@SiO2色谱柱时流动相中不同乙腈体积分数对磺胺类药物（a）分离效果和（b）保留因子的影响

为了进一步研究TAPT-TA-COF@SiO_2_微球材料填充色谱柱更多的分离性能，在相同条件下重复制备了该色谱柱。选择一系列化合物作为探针，在流动相为乙腈-水（30∶70、15∶85或40∶60，体积比）、柱温为30 ℃、流速为1 mL/min的色谱条件下，测试其在TAPT-TA-COF@SiO_2_色谱柱上的分离性能。如图8a所示，首先使用甲苯、苊、蒽、菲、芘混合物研究测试了TAPT-TA-COF@SiO_2_色谱柱对多环芳烃的分离性能。实验表明该类化合物在TAPT-TA-COF@SiO_2_柱上分离时疏水性越强其保留因子越强。接着，使用代表性碱性化合物包括苯胺、氯苯胺和萘胺以及甲酰胺类化合物测试TAPT-TA-COF@SiO_2_色谱柱分离性能，如图8b和8d所示，可以看出均实现了有效分离，且1-萘胺因疏水性最强在TAPT-TA-COF@SiO_2_柱上的保留时间最长。因为邻苯二甲酸酯是应用最广泛的增塑剂之一，对人体健康具有显著的毒副作用，其分析测定至关重要，本文进行了邻苯二甲酸酯类化合物的分离实验，如图8c所示，结果表明此类分析物在8 min内也可实现基线分离，出峰顺序与疏水性一致^［37］^。

**图8:**
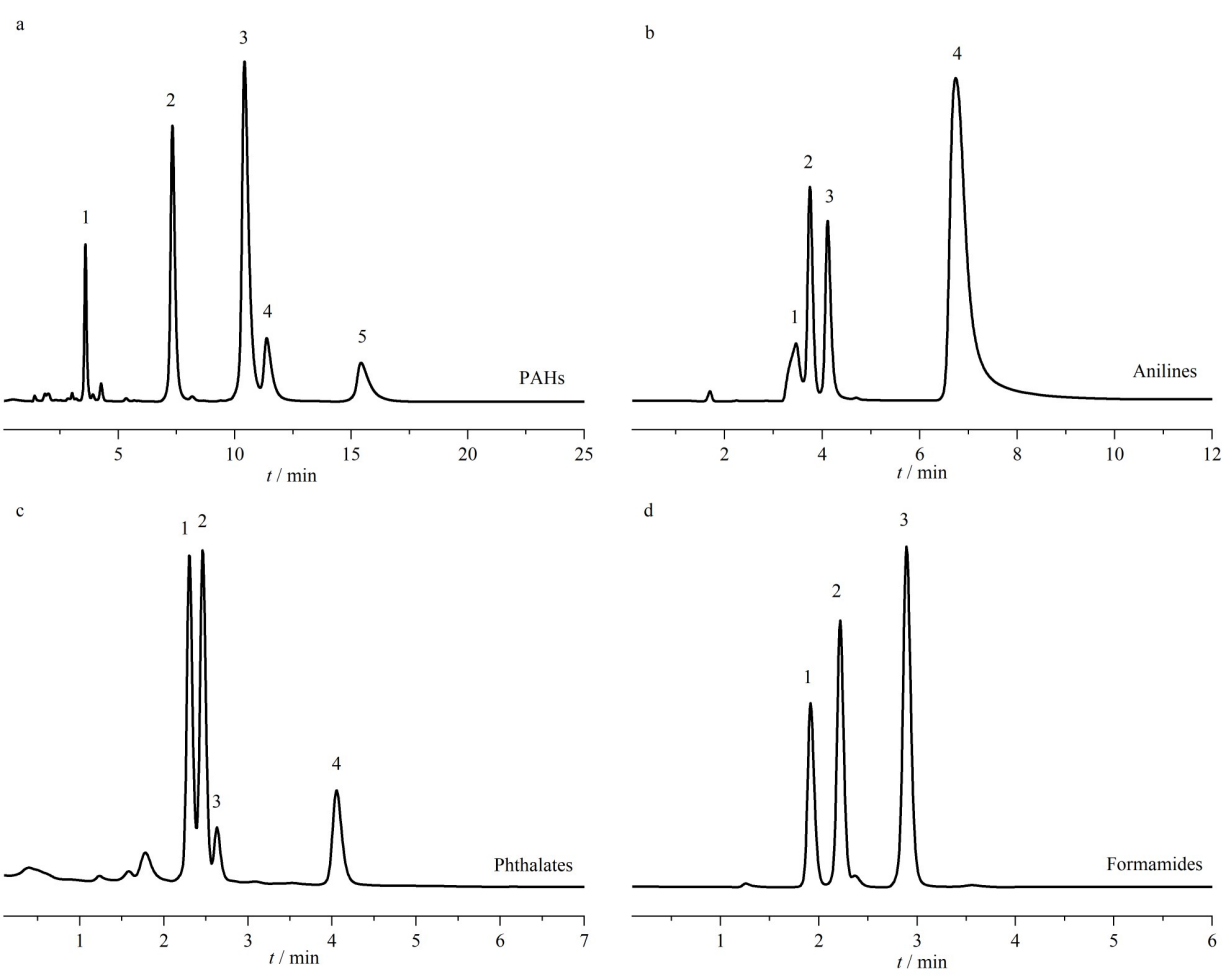
采用TAPT-TA-COF@SiO_2_色谱柱时（a）多环芳烃、（b）苯胺、（c）邻苯二甲酸盐和（d）甲酰胺类化合物的分离色谱图

### 2.3 TAPT-TA-COF@SiO_2_色谱柱的实际应用

#### 2.3.1 线性范围、精密度、稳定性和重复性

取黄芪甲苷标准品分别配制成质量浓度为0.5、1、1.5、2、2.5、3 mg/mL的标准溶液。分别吸取20 μL，平行进样2次，取平均值。以指标成分的峰面积对数为纵坐标（*y*），质量对数为横坐标（*x*），进行线性回归，得线性方程：*y*=1.559*x*+5.803，相关系数*r*=0.998 9，黄芪甲苷在10~60 μg范围内具有良好线性。按信噪比*S/N=*3和10计算检出限（LOD）和定量限（LOQ），分别为1 μg和3.3 μg。

取同一供试品溶液，连续进样6次，其峰面积RSD=1.34%，表明仪器精密度良好。取同一供试品溶液，分别在制备后0、2、4、8、12、24 h时测定其峰面积，其峰面积RSD=1.73%，表明供试品溶液在24 h内基本稳定。精密称取同一批黄芪样品6份，按供试品溶液的制备方法进行备样，测得黄芪甲苷的平均质量分数为0.085%，RSD为2.08%。

#### 2.3.2 加样回收率试验

精密称取已知黄芪甲苷含量的黄芪样品约1 g，共9份，平均分为3组，按照低、中、高浓度分别加入黄芪甲苷对照品0.5、1.0、1.5 mg，按供试品溶液的制备方法进行备样，测定黄芪甲苷的含量，并计算回收率及RSD，结果见表2。

**表2:** 黄芪样品中黄芪甲苷的加标回收率（*n*=3）

No.	Background/mg	Added/mg	Found/（g/L）	Recovery/%	RSD/%
1	0.85	0.5	1.316	93.3	1.43
2	0.85	1.0	1.799	94.9	1.71
3	0.85	1.5	2.333	98.8	1.25

#### 2.3.3 实际样品检测

取不同批次的黄芪样品粉末，分别按1.3.3节方法制备供试品溶液，按1.3.4节色谱条件进样20 μL，得到实际样品色谱（见图9），然后用外标两点法计算含量，结果表明黄芪中黄芪甲苷的含量为0.085%。本研究采用了改进的水提取方法与C18固相萃取处理样品，相比较山西大学之前报道的采用甲醇提取及正丁醇萃取方法^［4］^，减少了有机溶剂，并且简化了处理过程。

**图9:**
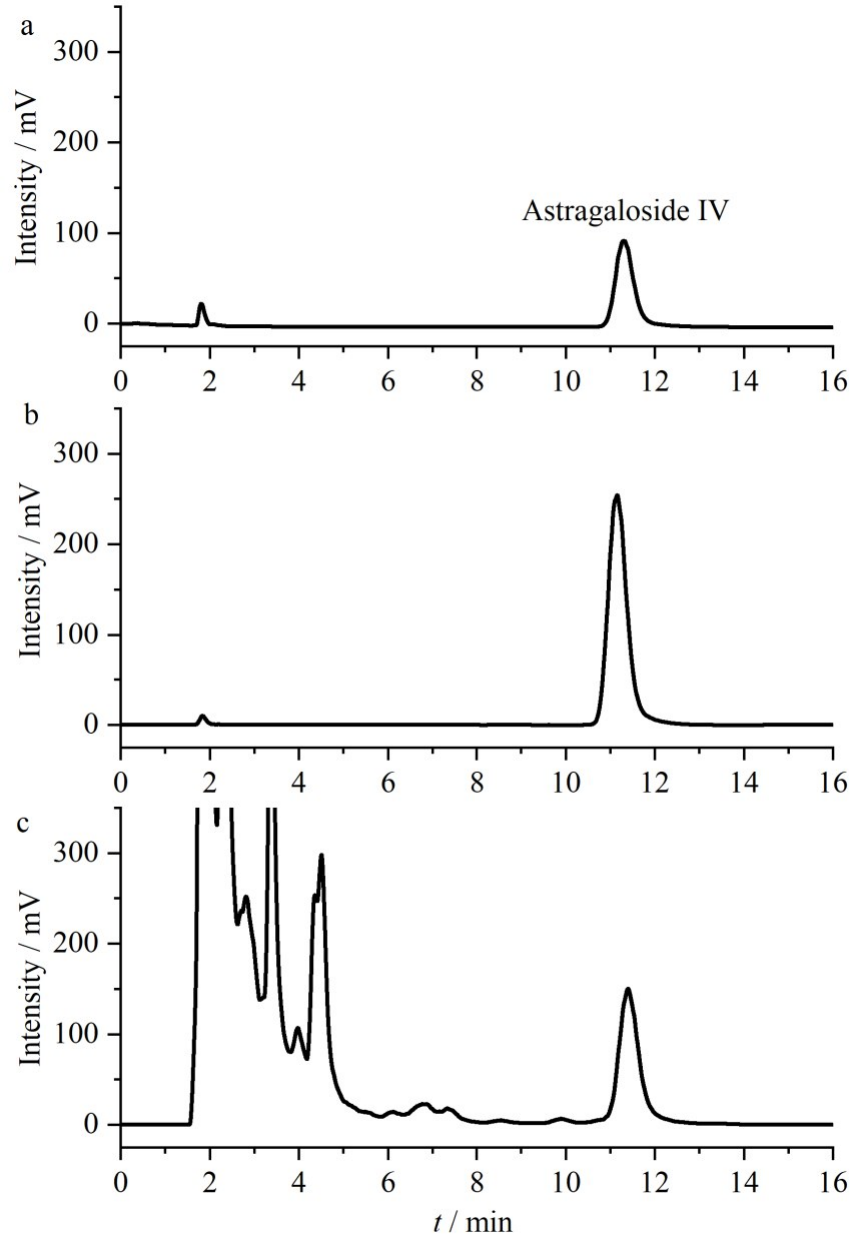
黄芪甲苷的（a、b）对照品和（c）实际样品色谱图

## 3 结论

本研究以合成的SiO_2_为载体逐层生长了纳米COFs，制备了亚胺连接TAPT-TA-COF@SiO_2_介孔核壳固定相。新制备的TAPT-TA-COF@SiO_2_色谱柱由于反相保留机制对取代苯、烷基苯、邻苯二甲酸酯、甲酰胺化合物、苯胺等化合物展现了优异的分离度和色谱柱效率。本工作与前面工作的不同点如下：第一，将共价有机骨架材料均匀涂覆于二氧化硅表面，未过多改变原有介孔二氧化硅的孔径和比表面积。第二，该材料是一类很有潜力的分离固定相，在中药标志性化合物含量测定、分离纯化方面将有很大应用前景。因此，本工作进一步推进了COF功能材料在色谱柱中的分离应用。未来该材料可以用来处理中药水提液，优化对中药材进行多指标成分的含量测定，推动中药质量评价研究。此外，未来可选择更多种类单体，发展制备新型亲水型、离子型、手性单体组成的共价有机骨架固定相且研究更多中药化合物的分离应用，以及生物医药材料、环境分析等领域应用。
